# Simulated Raman correlation spectroscopy for quantifying nucleic acid-silver composites

**DOI:** 10.1038/srep23535

**Published:** 2016-03-24

**Authors:** Lindsay M. Freeman, Alexei Smolyaninov, Lin Pang, Yeshaiahu Fainman

**Affiliations:** 1 Department of Electrical and Computer Engineering, University of California San Diego, 9500 Gilman Drive, La Jolla, California 92093-0407, USA.

## Abstract

Plasmonic devices are of great interest due to their ability to confine light to the nanoscale level and dramatically increase the intensity of the electromagnetic field, functioning as high performance platforms for Raman signal enhancement. While Raman spectroscopy has been proposed as a tool to identify the preferential binding sites and adsorption configurations of molecules to nanoparticles, the results have been limited by the assumption that a single binding site is responsible for molecular adsorption. Here, we develop the simulated Raman correlation spectroscopy (SRCS) process to determine which binding sites of a molecule preferentially bind to a plasmonic material and in what capacity. We apply the method to the case of nucleic acids binding to silver, discovering that multiple atoms are responsible for adsorption kinetics. This method can be applied to future systems, such as to study the molecular orientation of adsorbates to films or protein conformation upon adsorption.

Silver nanoparticles functionalized with nucleic acids are unique platforms offering beneficial optical properties that can be exploited for bioimaging and sensing applications, including generation of fluorescence^1^,^2^,^3^,^4^,^5^ and enhancement of Raman signals^6^,^7^,^8^,^9^. The electron transfer between silver and nucleic acids creates electronic transitions that induce a chemical resonance enhancement for surface-enhanced Raman spectroscopy (SERS)^10^,^11^,^12^,^13^, a valuable tool for molecular analysis that detects vibrational fingerprints^14^,^15^,^16^. The specific atoms of the nucleic acids (e.g. nitrogen atom N1 or N3 of adenine) that bind to the silver nanoparticles significantly affect the generated chemical resonance enhancement as the charge-transfer between the nucleic acids and silver modulates the electronic transitions and the molecular configuration induces a change in the polarizability. Therefore, determining the preferential binding of atoms and molecular orientation of nucleic acids interacting with the silver surface assists with characterizing the optical system properties and enables a better understanding of the physical phenomena of enhanced fluorescence and Raman signals via DNA coated silver particles. Previously, researchers have attempted to identify the atomic binding sites via x-ray absorption^17^, surface-enhanced Infrared spectroscopy (SEIRS)^18^, scanning tunneling microscopy (STM)^19^, and SERS^20^,^21^. However, these methods primarily rely on qualitative analysis rather than quantitative correlation calculations and also assume that only one binding atom is responsible for the chemical bonding. Here, we quantitatively determine the preferential binding sites of each nucleic acid to silver by developing a simulated Raman correlation spectroscopy (SRCS) method, demonstrating experimentally that multiple atoms are responsible for molecular bonding and calculating the composition of the binding sites to quantify these effects. The unique Raman signatures of the various potential binding sites are calculated using a time-dependent density functional theory (TD-DFT) method^22^ and the simulated results are statistically analyzed and compared to experimental measurements in order to deduce the probabilities of the binding sites. The correlation coefficient between simulated and experimental Raman spectra is optimized to be greater than 0.8 by varying the composition of binding atoms to the silver surface until a maximum is found, revealing that nucleic acids have a mixture of preferential binding atoms to silver. The SRCS process for binding site determination can be applied to other research areas of interest, including quantitative analysis of tip-enhanced Raman scattering (TERS) to distinguish the orientation of molecules with respect to a surface^23^ and SERS detection of protein conformation to metal films^24^.

By attaching nucleic acids to silver nanoparticles or other plasmonic structures, the optical properties of such systems can be tuned in order to modify the function of these systems. Thus, certain characteristics can be either attenuated or amplified by understanding and controlling the way in which nucleic acids bind to the surface, as experienced when detecting the chirality of DNA strands grown on silver nanocrystals^5^ and controlling the plasmonic properties of silver nanoparticles incubated with nucleic acids^9^. While there have been reports of finding the preferred binding sites of nucleic acids to silver, such as adenine binding to silver via the amino group^25^, N1^20^, N3^26^, or N7^27^ atoms, the reports are often inconclusive, contradictory, and limited to qualitative analysis. There exists a need to quantitatively analyze the multiple potential binding sites of nucleic acids to silver, while also exploring the favorable optical properties that such systems provide. The Raman signal, which is determined by the molecular structure of the nucleic acid and by slight changes to bond lengths and angles, can yield information regarding the stress and strain the system undergoes when certain atoms bind to the metal. When nucleic acids bind to silver, the orientation of the molecule with respect to the metal surface determines the polarizability of the system, leading to either enhanced or reduced Raman signal frequency modes depending on the molecular orientation with respect to the silver surface^28^. Therefore, by analyzing the calculated Raman signals of various atoms of nucleic acids binding to silver and comparing to experimental measurements, the preferred binding composition can be found and the nucleic acid silver system can be characterized quantitatively. Here, we develop the SRCS method to quantitatively compare simulated Raman spectra with experimental measurements, demonstrating the ability to discover and quantify the composition of binding sites of nucleic acid-silver composites. We calculate the composition of binding sites for each nucleic acid-silver composite and measure the correlation of the superimposed simulated Raman spectra to the experimental Raman spectrum. Thus, we have proven that multiple binding sites are responsible for adsorption within nucleic-acid silver composites and implemented a statistical based method to characterize such systems.

## Results

**Figure 1:**
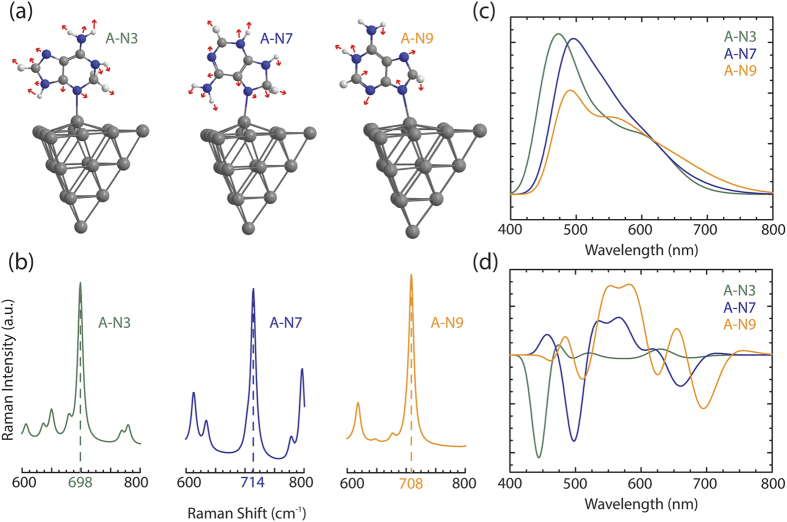
Variation of optical signals dependent on binding sites of adenine (A). (**a**) Optimized geometrical structures for 3 systems: A-N3 (green), A-N7 (blue), and A-N9 (orange) using TD-DFT calculations. The red arrows demonstrate the force displacement vectors for the ring-breathing-mode. (**b**) Simulated Raman spectra of the three systems for the ring-breathing-mode. The geometrical strain on the systems cause slight shifts in the location of Raman modes. (**c,d**) UV-VIS absorption (**c**) and circular dichroism (**d**) simulated spectra for the 3 systems, demonstrating modulation of absorption dependent on binding site.

To determine the molecular orientation and polarizability, we use TD-DFT to geometrically optimize the structures and calculate the electronic transitions and vibrational frequencies. The geometry optimization repositions the atomic structure of the system to reach an energy minimum, the electronic transition calculations apply an external potential and compute the change in charge density, and the vibrational frequency calculations vary the induced energy of the system and output the coordinates of the atomic vibrations for each frequency mode. As an example, depending on the adenine atom (N3, N7, or N9) that binds to the surface of a 20 atom silver tetrahedral surface (Fig. 1a), the simulated Raman signal (Fig. 1b), ultraviolet-visible absorption (Fig. 1c) and circular dichroism (Fig. 1d) spectra demonstrate differing results due to the change in the polarizability, density of states and molecular orbitals of the systems. Although the UV-VIS absorption spectra are fairly similar, there are small changes in the intensity of the oscillator strength and bandwidth of each system. Circular dichroism spectroscopy simulations, used to study the chirality of molecules with polarized light, indicate significant changes in the optical properties caused by the change in geometrical structure and electron density of the systems. Thus, nucleic acids functionalized to silver induce distinct and notable transformations to the optical characteristics compared to isolated nucleic acids or silver nanoparticles.

**Figure 2:**
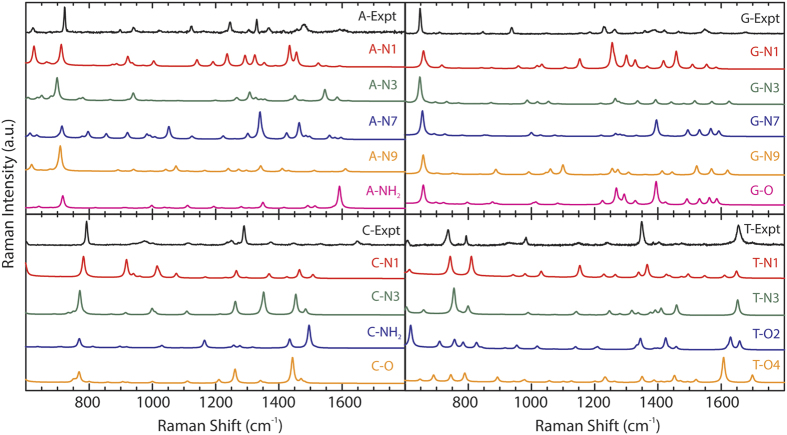
Experimental and simulated Raman spectrum for the 18 nucleic acid systems under study. Experimental Raman spectrum measurements (black) of the 4 nucleic acids (A - adenine, C - cytosine, G - guanine, T - thymine) attached to silver and simulated Raman spectra of binding atoms (red, green, blue, orange, magenta) of the 18 systems under study.

To harness the remarkable optical properties of the nucleic acid-silver composites, the accurate binding site compositions must be found and the systems appropriately characterized. The data for the SRCS process is acquired by experimentally measuring the Raman signatures of the four nucleic acids functionalized to silver and calculating the simulated Raman spectra for 18 systems of nucleic acids bound to silver via potential binding atoms (Fig. 2). By considering every possible tautomer of each nucleic acid found in water^29^,^30^,^31^,^32^, there are 18 potential nucleic acid-silver configurations that undergo TD-DFT geometrical optimization and Raman frequency simulations (5 for A-Ag, 4 for C-Ag, 5 for G-Ag, and 4 for T-Ag; Supplementary Section 1). To directly compare with the simulated 18 nucleic acid silver configurations, nucleic acids are dissolved in water for experimental Raman studies of samples in order to permit multiple tautomers to potentially bind to the silver surface. The silver substrates used for experimental measurements are random silver films fabricated in an electron beam evaporator^33^, adopting the face-centered cubic (111) surface^34^ and demonstrating a consistent electromagnetic field enhancement effect at an excitation wavelength of 785 nm. The extinction spectra of random silver films with thicknesses of 30 nm have shown to have weak to non-existent surface plasmon resonance at excitation wavelengths of 785 nm^35^, so the electromagnetic enhancement does not factor into the experimental Raman spectra (Supplementary Section 2). As studied previously, the surface of a 20 atom silver tetrahedral surface is the model system to use in TD-DFT simulations when representing the fcc (111) surface^36^, and thus random silver films are the ideal substrate to use for our experimental measurements. For statistical analysis, the intensity of the Raman frequency modes are used rather than the location of the Raman frequency modes due to the incomplete basis set and neglect of anharmonicity^37^. The prominent Raman frequency modes are assigned discrete values by visualizing each mode in GaussView (Supplementary Section 3).

When comparing the simulated spectra to the experimental spectrum for each nucleic acid (NA), we conclude that single binding site simulations have deviations with respect to the experimental measurement, as determined by linear regression analysis, and thus the experimental measurement cannot be deduced to a single binding site (Supplementary Section 4). Thus, SRCS is employed to determine the weighted composition of binding sites by optimizing the calculated correlation. The SRCS process for each nucleic acid consists of 1) assigning the continuous Raman frequency spectral modes to discrete values for the experimental spectrum and all simulated binding site spectra; 2) normalizing the Raman intensity of each frequency mode with respect to the sum of the Raman modal intensities of the selected Raman modes for each spectrum, and 3) calculating the weighted coefficients for each binding site that yield the optimal coefficient of determination in terms of all binding sites, *r*^*2*^, when comparing the cumulative weighted simulated Raman spectra to the experimentally detected Raman spectrum.

We consider the case of a nucleic acid that has an experimental Raman spectrum, 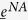, and multiple corresponding simulated Raman spectra, 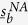, in which the potential binding sites are described as b = 1, 2, …, B and the discrete frequency modes as m = 1, 2, …, M. The discrete frequency modes are extracted from the continuous Raman spectra 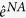 and 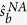, in which the GaussView program is used to visualize and assign the frequency modes to discrete values (Supplementary Section 3). We denote the modal intensities of the experimental measured spectra 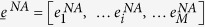 and the modal intensities of the simulated spectra 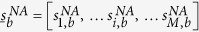, in which b designates the binding site (e.g., N1, N3). Then, the experimental Raman intensity of the frequency mode *i* is normalized to 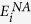 with the following equation:


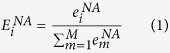


in which 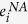 is the experimental Raman intensity at a specific frequency mode *i*, 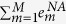 is the sum of the experimental Raman intensities of frequency modes from 1 through M, and 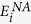 is the normalized experimental Raman intensity for mode *i*. The normalized modal intensities for the experimental Raman measurement can be designated as:


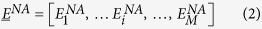


For the case of the simulated Raman intensities, assuming the simulated spectrum is for a nucleic acid bound to silver via the binding site *b*, the normalized Raman intensity for mode *i* is represented by the following equation:


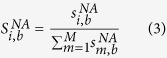


in which 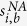 is the simulated Raman intensity of a nucleic acid bound via binding site *b* at a specific frequency mode *i*, 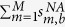 is the sum of the simulated Raman intensities of a nucleic acid bound via binding site *b* from modes 1 to *M* and 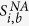 is the normalized simulated Raman intensity mode of a nucleic acid bound via binding site *b* for mode *i*. Thus, the normalized modal intensities for a simulated Raman measurement can be designated as:


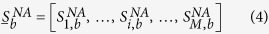


Now, given the experimental discrete Raman mode intensities 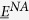, we assume that there is a set of corresponding weighted constants that maximize the correlation of 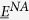 with respect to a linear superposition of a set of weighted spectra 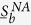, where b = 1,2,…B. We define the weighted superimposed simulated spectra 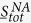 as:


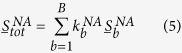


where each value of 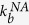 represents the weighted constants for the corresponding simulated spectrum based on binding site b. The weighted constants are normalized as:


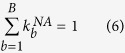


Then, we vary the values of 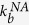 in equation 5 in order to optimize the correlation between the weighted spectral modes 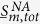 of the simulated data 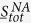 and the measured spectral modes 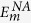 of the experimental data 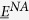 by using the following equation for the coefficient of determination:


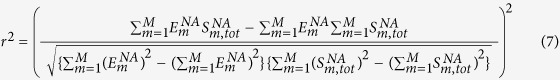


**Figure 3:**
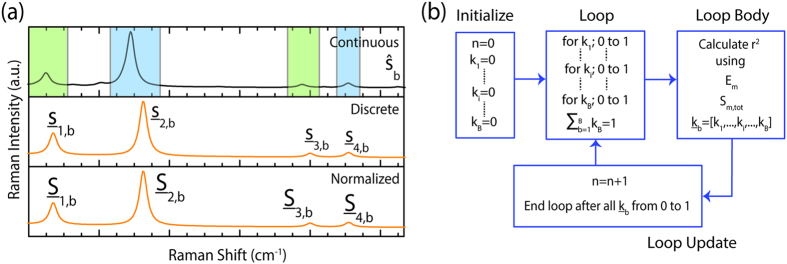
Discrete mode analysis and schematic diagram of SRCS. (**a**) The normalization procedure for each spectrum, in which the continuous spectrum 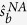 (top) is assigned discrete modal values m = [1,2,…,M] and aligned with the corresponding experimental discrete modes 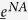, described as a vector 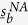 (middle). The Raman intensity of each mode is then normalized using equation 3 to generate 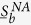 (bottom). **(b)** Flow diagram describing the SRCS process, in which the values of 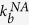 are varied while calculating *r*^2^ using 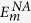 and 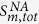 given by equation 2 and equation 5, respectively.

The first two steps of the SRCS method are shown in Fig. 3a, in which the continuous Raman spectrum 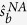 is assigned discrete modes 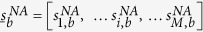 and then normalized to generate 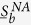. The value of *r*^2^ is optimized for the case of adenine by varying the weighted constants of 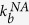 in equation 5 and calculating the value of 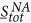 (Fig. 3b). The coefficient of determination given by *r*^2^ measures the linear correlation between an experimental spectrum 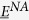 and weighted calculated spectrum 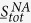, with *r*^2^ = 1 corresponding to perfect correlation. By applying signal processing techniques^38^, the weighted constants 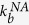 which produce an 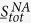 that maximizes *r*^2^ are calculated and represent the best possible compositional analysis of experimental binding sites within the limits and numerical accuracy of the simulated binding sites and determine how well the chosen weighted spectra works as a least squares estimator for the experimental spectrum.

**Figure 4:**
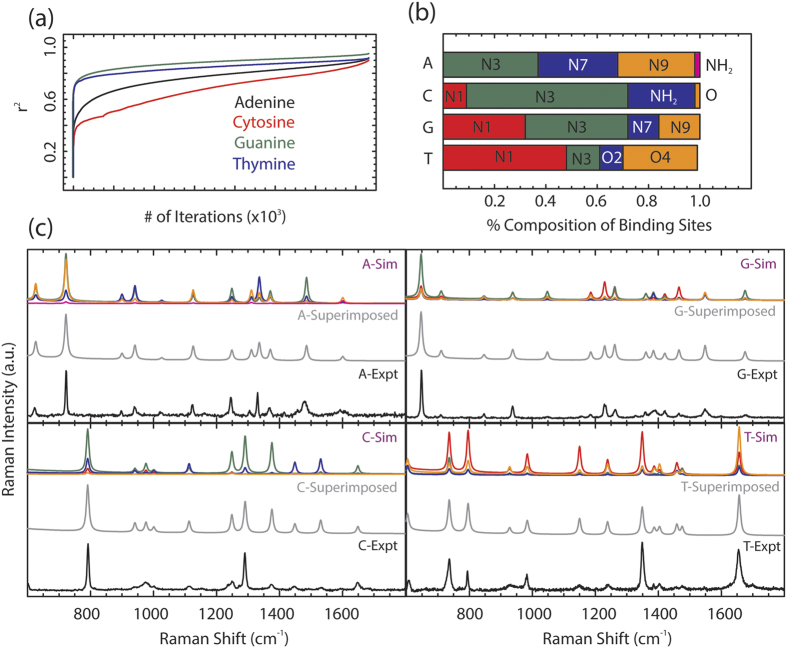
Optimizing the coefficient of determinations with weighted correlation method. (**a**) The increase in the coefficient of determination (*r*^*2*^) as the number of iterations increases, resulting in *r*^*2*^ near 1, for the nucleic acids adenine (black), cytosine (red), guanine (green), and thymine (blue). (**b**) The optimized percent composition of each binding site to silver at the maximum coefficient of determination for each nucleic acid. Adenine shows the majority of binding comes from N3, N7, and N9. Cytosine is primarily bound to silver via the N3 atom. Guanine’s strongest binding atoms are N1 and N3. Thymine is strongly bound via the N1 and O4 atoms. (**c**) The weighted simulated Raman spectra 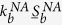 for each binding site (top in A, C, G and T; multicolored), the superimposed simulated Raman spectra 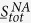 for all the binding sites (middle in A, C, G and T; gray), and the experimental measured Raman spectra 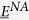 for each nucleic acid (bottom in A, C, G and T; black).

The SRCS process is applied to all four nucleic acids, in which approximately 175,000 iterations of varying 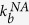 to calculate 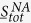 and optimize *r*^2^ is conducted per nucleic acid (Fig. 4a), determining the optimized binding site compositions for each nucleic acid (Fig. 4b, Table 1). To qualitatively compare the effectiveness of the SRCS process, the individual weighted binding site Raman spectra 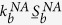, the superimposed cumulative simulated Raman spectrum 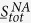, and the experimental Raman spectrum 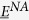 are plotted (Fig. 4c). When comparing the superimposed simulated Raman correlation spectrum to the experimental measurement, qualitative analysis clearly shows that the weighted composition calculated spectrum (gray) for each nucleic acid is in good agreement with the experimental Raman signature (black).

The high coefficients of determination and corresponding superimposed spectra demonstrate the efficiency of the SRCS process as a method to discover the preferential binding sites of each nucleic acid to silver. The preferential binding sites of adenine to silver are N3, N7, and N9, with limited binding from NH_2_ and none from N1. For cytosine, the preferred binding site is N3 with moderate binding from NH_2_ and limited binding from N1 and O. The results of guanine demonstrate that there is no binding via the O atom, with moderate bonding from N1 and N3 and limited binding from N7 and N9. Finally, the N1 atom of thymine demonstrates the strongest binding to silver with roughly half of the molecules binding via the N1 site, with moderate bonding from O4 and limited bonding from N3 and O2. To explain the preference of specific binding sites to nucleic acids, we examine the physical existence of nucleic acid tautomers in water. Because nucleic acids exist as several tautomers upon interaction with water, there are a significant number of potential binding atoms available to bind to silver depending on the location of the hydrogen atom for each tautomer. The tautomers determine the available binding sites, such as when the N9H adenine tautomer enables the doubly bonded ring nitrogens to act as the preferential binding sites and electron donors, while prohibiting the N9 atom from binding to the silver surface. Thus, the composition of the preferential binding atoms is dependent on the prevalence of specific tautomers of the nucleic acids in water. For example, in the case of cytosine, it can be deduced that the N3H tautomer is present in a limited form in water because of the dominance of the N3 and NH_2_ as binding sites. This agrees well with a previous study, which determined that upon cytosine hydration, the N1H tautomer formed much more frequently than the N3H tautomer, enabling the doubly bonded ring nitrogen group on N3 to bind, while prohibiting the N1H site to bind to silver^30^. Therefore, in addition to determining the composition of binding sites of nucleic acids to silver, the SRCS method can yield insight into the population of specific tautomers in water. Additional factors that can determine preferential binding sites are the electron densities of the molecules and the geometrical constraints caused by steric effects and will be addressed in future work.

In this paper, we have developed and applied an SRCS statistical analysis method for determining the composition of binding atoms of nucleic acids to silver using a quantitative statistical analysis process that compares calculated Raman spectra to experimental measurements. We have demonstrated that the SRCS method can deduce the relative composition of binding sites and quantitatively determine that certain sites (e.g. N1 in adenine, O in cytosine) do not play a significant role in binding to silver, while others (e.g. N3 in adenine, N3 in cytosine), are the primary binding sites with additional, less active binding sites (e.g. N7 and N9 in adenine, N1 in cytosine). Using a weighted simulated Raman correlation spectroscopy method, we have developed a process to optimize the coefficient of determination for comparing simulated Raman data to experimental measurements by adjusting the composition of binding sites. By optimizing the composition of the potential binding sites, we show that a mixture of orientations and configurations occur during experimental measurements and provide a method to use for binding analysis. Additionally, we demonstrate the efficiency of our method by comparing the experimental measurements to the superimposed simulated Raman spectra, showing excellent agreement and a high coefficient of determination. Finally, we compare the composition of binding atoms to previously published results, justifying that this method can be used to estimate the molecular dynamics of nucleic acids. This method lays the foundation for using computational simulations to study the assembly and surface chemistry of molecules on the nanoscale level and can be applied to many fields of study, including near-field characterization of molecule alignment and configuration of molecules with respect to metal surfaces.

## Methods

### Time-dependent density functional calculations of nucleic acid-silver complexes

TD-DFT is performed using the Gaussian 09 package^22^ for each potential binding site of the four nucleic acids to a 20 atom silver tetrahedral structure (Ag20), resulting in 18 unique systems for our study. The complete list of systems and their corresponding atomic labels can be found in supplementary section 1. The nucleic acids bind to the surface (S), the vertex (V), or the edge (E) of the Ag20 face-centered cubic (fcc) lattice structure. The preferred binding site of the Ag20, as previously reported^39^, is the flat surface and the majority of the nucleic acid orientations prefer that site. However, there are some nucleic acid silver (NA-Ag) systems which are more stable when binding to the edge of the structure.

The TD-DFT calculations were performed using Gaussian 09^22^ software on the Gordon supercomputer at the University of California, San Diego^40^. All 18 structures are geometrically optimized using the B3LYP method^41^ and LANL2DZ^42^ basis set. B3LYP is chosen as the density functional theory method as it uses both generalized gradient approximations and the local-density approximation and has been shown to be an accurate model for molecules attached to the Ag20 structure. LANL2DZ is used to account for the silver atoms. A very tight convergence criterion was used and a super fine grid of 150,974 and 225,974 was required for the nucleic acid atoms and silver atoms, respectively. Once optimized, each structure is confirmed to have no negative frequency, showing successful convergence.

The electronic transitions and Raman frequencies are calculated via Gaussian 09, using the same basis set and method as the geometric optimization. TD-DFT calculates Raman optical activities for each mode, which are converted to Raman intensities using the following relationship^43^:


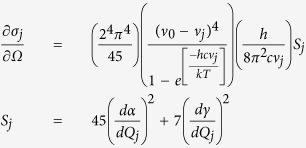


where σ is the Raman cross-section, Ω is the depolarization ratio, ν_0_ is the frequency of the incident light, ν_j_ is the frequency of the vibrational mode, S_j_ is the Raman scattering factor, α is the polarizability, γ is the anisotropic polarizability, and Q_j_ is the normal coordinate. An incident wavelength of 785 nm and a temperature of 293 K are used to match experimental conditions. The simulated Raman spectra is plotted by applying a Lorentzian fit for each Raman intensity mode, using a total of 3000 points between 400 cm^−1^ and 4000 cm^−1^ with a full-width half maximum of 10 cm^−1^. Because TD-DFT overestimates the location of the frequency mode due to the neglect of electron correlation and anharmonicity^44^, a scaling factor of 0.9891 for 600 cm^−1^ to 1000 cm^−1^ and of 0.9568 for 1000 cm^−1^ to 1800 cm^−1^ is used, as determined for the B3LYP method and LANL2DZ basis set^45^. Simulated Raman spectra are obtained for each of the 18 systems.

### Experimental Raman measurements of nucleic acids

For the experimental nucleic acid Raman data, silver films are fabricated by depositing 300 Å of silver using an e-beam evaporator (Temescal BJD, UCSD Nano3 Cleanroom) on a silicon wafer. The scanning electron beam (SEM) image of the surface films can be found in supplementary section 2. Nucleic acids are deposited at a concentration of 1 mM and are incubated on the silver films overnight. Before measuring the Raman spectra, the samples are rinsed with H_2_O to remove any large particles. The Raman measurements are acquired using a Renishaw Raman spectrometer at a wavelength of 785 nm. The sample is imaged using a 40x objective and the spectra are recorded with the hyperSpec program. A 60 s acquisition time is used with a Raman spectral range of 550–2000 cm^−1^. Baseline subtraction is performed to ensure all spectra are aligned with the x-axis.

## Additional Information

**How to cite this article**: Freeman, L. M. *et al.* Simulated Raman correlation spectroscopy for quantifying nucleic acid-silver composites. *Sci. Rep.* **6**, 23535; doi: 10.1038/srep23535 (2016).

## Supplementary Material

Supplementary Information

## Figures and Tables

**Table 1:** Coefficients of determination for 18 single atom binding systems and the 4 optimized weighted composition binding site systems.

Binding Site	*r*^*2*^ of Adenine	*r*^*2*^ of Cytosine	*r*^*2*^ of Guanine	*r*^*2*^ of Thymine
N1	0.20	0.06	0.21	0.52
N3	0.73	0.71	0.78	0.44
N7	0.14		0.57	
N9	0.62		0.55	
NH_2_	0.02	0.16		
O2		0.11	0.28	0.45
O4				0.41
Weighted Mixture	**0.85**	**0.81**	**0.91**	**0.84**
Optimized Compositions	37% N3	9% N1	32% N1	49% N1
	31% N7	63% N3	40% N3	13% N3
	30% N9	26% NH2	12% N7	9% O2
	2% NH2	2% O	16% N9	29% O4

The coefficient of determinations for single binding sites can be found in Supplementary Section 4. The coefficient of determination increases when accounting for a mixture binding sites for each nucleic acid, such as thymine which went from a r^2^ value of 0.52 (T-N1) to a value of 0.84 (48% T-N1, 14% T-N3, 9% T-O2, 29% T-O4).
