# Supplementary material for: Simulated Raman correlation spectroscopy for quantifying nucleic acid-silver composites
Source: Sci Rep. 2016 Mar 24;6:23535. doi: 10.1038/srep23535 (PMC4806353; doi:10.1038/srep23535)
Supplement: Supplementary Information [file srep23535-s1.pdf]

Simulated Raman correlation spectroscopy for quantifying nucleic acid-silver composites

Lindsay M. Freeman, Alexei Smolyaninov, Lin Pang, Yeshaiahu Fainman

Content of Sections

S1. Optimized geometry and vibration frequency analysis of adenine, cytosine, guanine, and thymine to silver

S2. Surface image and characterization of silver films

S3. Mode assignments for SRCS Process

S4. Coefficient of determination calculations for single binding Sites

S5. Optimized geometrical Cartesian coordinates for nucleic acid-silver composites

S1. Optimized geometry, molecular orbitals and vibration frequency analysis of adenine, cytosine, guanine and thymine to silver

The 20 atom silver tetrahedral model has been the standard TD-DFT model for the past 10 years when representing silver nanoparticles. The model has three potential binding sites: the surface (S), the vertex (V), or the edge (E). The surface and edge represent the face-centered cubic (111) lattice and the vertex represents the adatom site. Due to the size of the silver films, the adatom site is an unrealistic model for this system and thus will not be used. The nucleic acids can then bind to either the surface or the edge, with the surface being the common preferential binding site with the minimum energy geometry. The available binding sites for each nucleic acid are determined by the presence of the most stable tautomers of adenine, cytosine, guanine, and thymine in water.

S1.1. Geometrical optimization and vibrational frequency calculations of adenine

The potential binding sites of adenine to silver are N1 to surface, N3 to surface, N7 to surface, N9 to surface, and NH_2 / N7 to surface and edge, respectively (Figure S1).

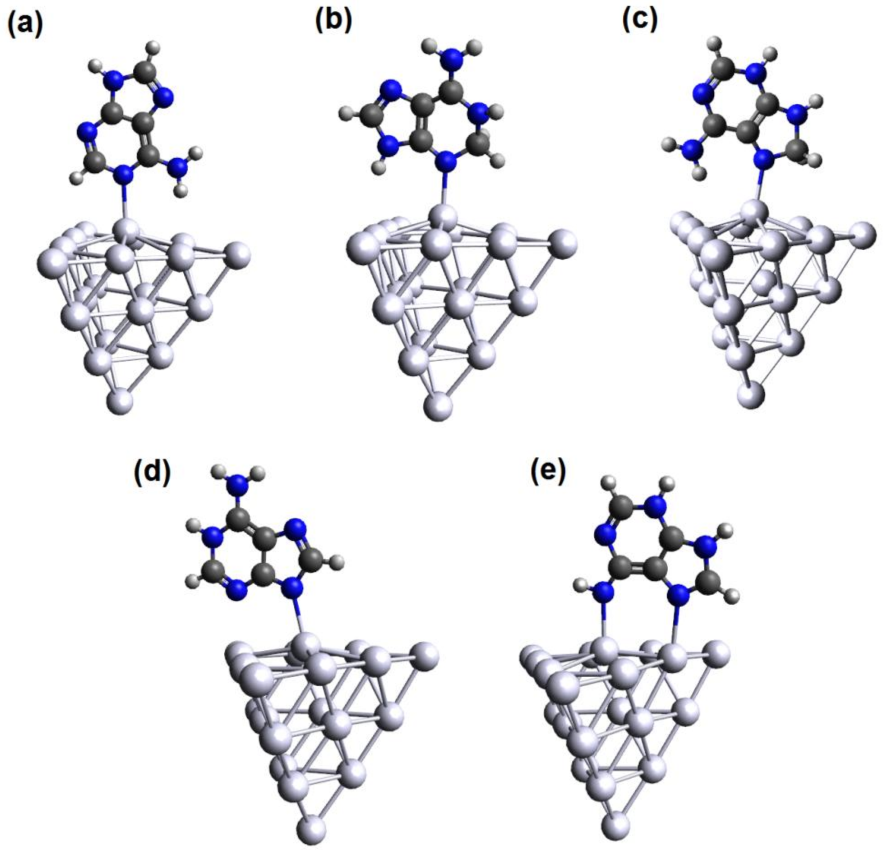

Supplementary Figure 1. Optimized geometries of adenine to the silver tetrahedral structure of (a) N1-S, (b) N3-S, (c) N7-S, (d) N9-S, and (e) NH_2-S,N7-E

The experimental and calculated frequency modes for each adenine system can be found in supplementary table 1. The top line of each frequency mode represents the location of the frequency and the bottom line represents the normalized Raman intensity, in which each mode intensity is normalized with respect to the total intensity of the spectrum.

Supplementary Table 1. Experimental and calculated frequency modes (top row of each mode) and intensity ratios (bottom row of each mode) of adenine on multiple binding sites

**Mode**	**Expt**	**N1**	**N3**	**N7**	**N9**	**NH_2**

**605-700 cm^{-1}**<br>def R5 (sqz group C5-N7-C8), R6 (sqz group C4-C5-C6, N1-C6-N10), rock NH_2	625	625-697	605-649	611-633	609-700	623-676
	0.0559	0.143	0.0892	0.0439	0.140	0.0352
**678-779 cm^{-1}**<br>ring-breathing mode	721	710	678-779	713	708	716
	0.300	0.135	0.322	0.0890	0.354	0.196
**777-935 cm^{-1}**<br>def R5 (sqz group N7-C8-N9, str C5-N7, C4-N9), R6 (sqz group N1-C2-N3), bend N9-H	899	886-935	853-921	777-796	890-914	819-854
	0.0303	0.0276	0.0211	0.0617	0.0239	0.00862
**853-991 cm^{-1}**<br>wag C2-H, def R5 (sqz group N7-C8-N9)	940	921	938	853-991	940	908-974
	0.0559	0.0615	0.0857	0.133	0.0296	0.0915
**969-1081 cm^{-1}**<br>rock NH_2, bend C2-H	1025	969	980-1081	1007-1040	993	997
	0.0224	0.00405	0.0147	0.0132	0.00575	0.0444
**1003-1264 cm^{-1}**<br>str C6-N10, N3-C4, C4-N9, bend N9-H, C2-H, N10-H11, C8-H	1125	1003-1046	1198-1264	1051	1041-1073	1037-1110
	0.0687	0.0352	0.0485	0.0951	0.108	0.0671
**1109-1306 cm^{-1}**<br>rock NH_2, str C5-N7, N1-C2, C2-N3, N7-C8, bend N9-H, C8-H	1248	1140	1306	1124-1259	1109-1165	1193-1219
	0.121	0.0439	0.0975	0.0494	0.0246	0.0372
**1190-1349 cm^{-1}**<br>Str C2-N3, C5-N7, N1-C2, C5-C6	1310	1190-1235	1326	1301	1272-1332	1280-1349
	0.0288	0.114	0.0306	0.0411	0.105	0.131
**1292-1416 cm^{-1}**<br>Str N1-C2, C6-N1, C4-C5, N3-C4, C5, N7, C8-N9, bend N9-H, C2-H, C8-H	1335	1292-1323	1342-1355	1340	1342	1416
	0.143	0.146	0.0327	0.207	0.0819	0.0134
**1352-1491 cm^{-1}**<br>str C5-N7, C6-N10, N1-C2, C6-N1, C8-N9, N3-C4, bend C2-H, N9-H, N10-H12, C8-H	1370	1352-1388	1437-1475	1349-1424	1410-1430	1491
	0.0440	0.0284	0.0804	0.0470	0.0602	0.0430
**1433-1572 cm^{-1}**<br>sciss NH_2, bend C2-H,	1485	1433-1454	1545-1583	1463-1497	1511	1514-1572

N9-H, str C6-N10, C6-N1, C2-N3, N3-C4, C4-C5, C8-N9, N1-C2	0.0967	0.225	0.173	0.0553	0.0199	0.0479
**1524-1619 cm^{-1}** sciss NH_2, str C6-N10, C5-C6	1600	1524-1592	1619	1559-1596	1567-1610	1591
	0.0335	0.0358	0.00528	0.0553	0.0469	0.357

S1.2. Geometrical optimization and vibrational frequency calculations of cytosine

Upon geometrical optimization, cytosine has 4 potential binding sites to the 20 atom silver tetrahedral structure: N1 to surface, N3 to edge, NH_2 to surface and O to surface as shown in Figure S2.

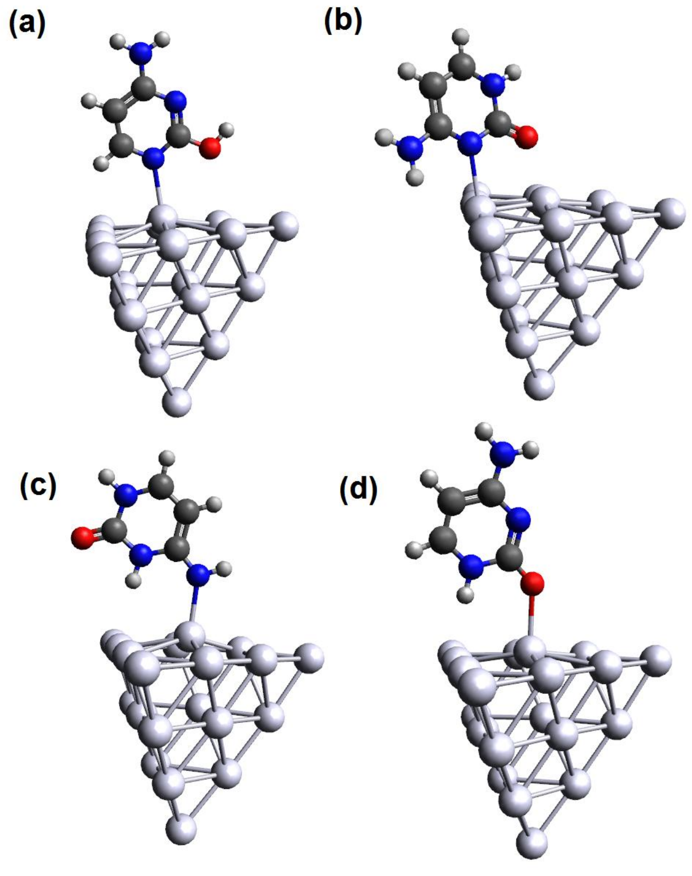

Supplementary Figure 2. Optimized geometries of cytosine to the silver tetrahedral structure of (a) N1-S, (b) N3-E, (c) NH_2-S, and (d) O-S

The experimental and calculated frequency modes for each cytosine system can be found in supplementary table 2. The top line of each frequency mode represents the location of the frequency and the bottom line represents the normalized Raman intensity, in which each mode intensity is normalized with respect to the total intensity of the spectrum.

Supplementary Table 2. Experimental and calculated frequency modes (top row of each mode) and intensity ratios (bottom row of each mode) of cytosine on multiple binding sites

**Mode**	**Expt**	**N1**	**N3**	**NH_2**	**O**
**636-810 cm^{-1}** ring-breathing-mode	790	781	688-748	636-810	711-799
	0.358	0.232	0.264	0.220	0.257
**858-959 cm^{-1}** rock NH_2, str C2-N1, C2-N3	940	917-959	839-916	895-918	858-920
	0.0221	0.246	0.0242	0.0452	0.0393
**975-1021 cm^{-1}** def C5-H, C6-H, tor C5-C6	975	1014-1021	998	1018	998-1001
	0.0591	0.158	0.0560	0.0124	0.0266
**1000-1110 cm^{-1}** Bend C5-H, C6-H, tor C4-C5	1000	1075	1008	1029	1110
	0.0303	0.0498	0.0167	0.0444	0.0349
**1100-1209 cm^{-1}** bend C6-C5-H, str C6-N1	1112	1167	1100-1108	1164	1209
	0.0262	0.0128	0.0340	0.150	0.0589
**1213-1265 cm^{-1}** rock NH_2, bend N1-C6-H, str C6-N1	1248	1230-1265	1213-1261	1240	1261
	0.0720	0.0901	0.131	0.00773	0.234
**1256-1351 cm^{-1}** str C2-N3, str C4-NH_2	1290	1297	1343-1351	1256-1276	1341
	0.284	0.00707	0.229	0.0926	0.0430
**1316-1452 cm^{-1}** str C4-NH_2, bend C5-C6-H	1375	1368	1452	1316	1354
	0.0390	0.0507	0.190	0.0178	0.0103
**1425-1448 cm^{-1}** str C4-N3, C6-N1, C4-C5, bend C4-NH_2	1448	1425	-	1433	1442
	0.0302	0.0152	0	0.175	0.216
**1464-1530 cm^{-1}** Bend C2-O, str C4-NH_2, C4-N3, C5-C6, sciss NH_2	1530	1464	1470	1495	1470
	0.0215	0.0967	0.00448	0.224	0.0629
**1481-1648 cm^{-1}** sciss NH_2, str C2-O	1648	1507	1484	1516	1481
	0.0571	0.0420	0.0493	0.00953	0.0170

S1.3. Geometrical optimization and vibrational frequency calculations of guanine

Guanine has 5 potential binding sites as shown in Figure S3: (a) N1 to surface and O to edge, (b) N3 to surface, (c) N7 to surface, (d) N9 to surface and N3 to edge, and (e) O to edge

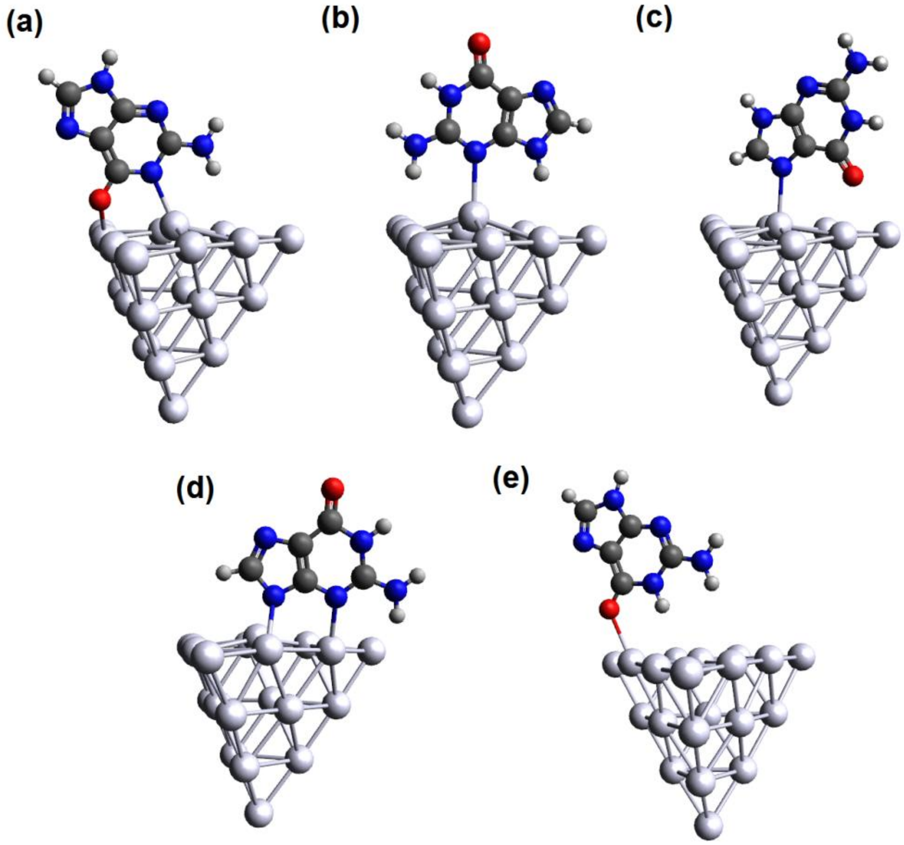

Supplementary Figure 3. Optimized geometries of guanine to silver tetrahedral structure of (a) N1-S and O-E, (b) N3-S, (c) N7-S, (d) N9-S and N3-E, and (e) O-E

The experimental and calculated frequency modes for each guanine system can be found in supplementary table 3. The top line of each frequency mode represents the location of the frequency and the bottom line represents the normalized Raman intensity, in which each mode intensity is normalized with respect to the total intensity of the spectrum.

Supplementary Table 3. Experimental and calculated frequency modes (top row of each mode) and intensity ratios (bottom row of each mode) of guanine on multiple binding sites

**Mode**	**Expt**	**N1**	**N3**	**N7**	**N9**	**O**
**646-696 cm^{-1}** ring-breathing-mode	646	658	646-669	654-667	657-665	657-696
	0.408	0.130	0.375	0.291	0.227	0.164
**692-797 cm^{-1}** def N3-C2-N1, N1-C6-O	710	716-769	692-769	692-780	699-764	698-797
	0.0243	0.0252	0.0597	0.0421	0.0303	0.0398
**839-907 cm^{-1}** def C4-N9-C8	846	839-873	842-883	850-907	836-887	852-889
	0.0300	0.0127	0.0248	0.0187	0.0619	0.0321
**937-1053 cm^{-1}** def N7-C8-N9	937	958-1032	983-996	998-1028	963-991	966-1013
	0.103	0.0776	0.0558	0.0554	0.0319	0.0318
**1045-1107 cm^{-1}** str C2-N1, C2-N3, C2-NH_2	1047	1107	1018-1053	1072-1094	1045-1059	1082-1094
	0.0186	0.0113	0.0680	0.0177	0.0790	0.0152
**1099-1224 cm^{-1}** def C8-H	1185	1152	1083-1092	1220	1099	1224
	0.0364	0.0781	0.00686	0.0127	0.111	0.0339
**1217-1268 cm^{-1}** def N1-H, str C8-N7, C8-N9, C6-N1, C5-N7	1229	1255	1218	1266	1217-1255	1268
	0.110	0.191	0.0134	0.0304	0.0738	0.138
**1261-1300 cm^{-1}** def C8-H, str C8-N9, C5-C6, C4-N9, C6-N1	1261	1300	1264-1288	1279-1291	1273	1293
	0.0314	0.104	0.112	0.0308	0.0551	0.0839
**1306-1360 cm^{-1}** str C2-N1, C4-N3, C5-C6	1360	1327	1335	1331	1306	1328
	0.0430	0.0667	0.0487	0.0103	0.0280	0.0328
**1360-1395 cm^{-1}** str C4-N9, C5-N7, C8-N9	1384	1366	1392	1395	1360	1394
	0.0430	0.0196	0.0629	0.192	0.00444	0.200
**1413-1442 cm^{-1}** def N1-H, str C2-N3, C5-C6, C8-N9	1420	1417	1442	1431	1413	1423
	0.0309	0.0625	0.0318	0.00425	0.0439	0.0175
**1444-1494 cm^{-1}** def C8-H, str C2-N1, C2-N3, C8-N7, C8-N9	1465	1457	1481	1494	1444	1491
	0.0308	0.138	0.00940	0.0736	0.0310	0.0503
**1509-1570 cm^{-1}** def N1-H, sciss NH_2, str C2-N2, C4-C5, C4-N9, C6-O, N3-C2, N3-C4	1548	1509-1553	1516	1531-1567	1522-1569	1531-1562
	0.0730	0.0707	0.0490	0.161	0.169	0.109
**1583-1675 cm^{-1}**	1675	1583	1570-1625	1592	1620	1586

sciss NH_2, str C2-N1, C2-N3	0.0178	0.0124	0.0826	0.0603	0.0532	0.0509

S1.4. Geometrical optimization and vibrational frequency calculations of thymine

Based on the most common thymine tautomers that exist in water, thymine has 4 potential binding sites as shown in Figure S4: N1 and O2 to edge, N3 and O2 to edge, O2 to edge, and O4 to edge

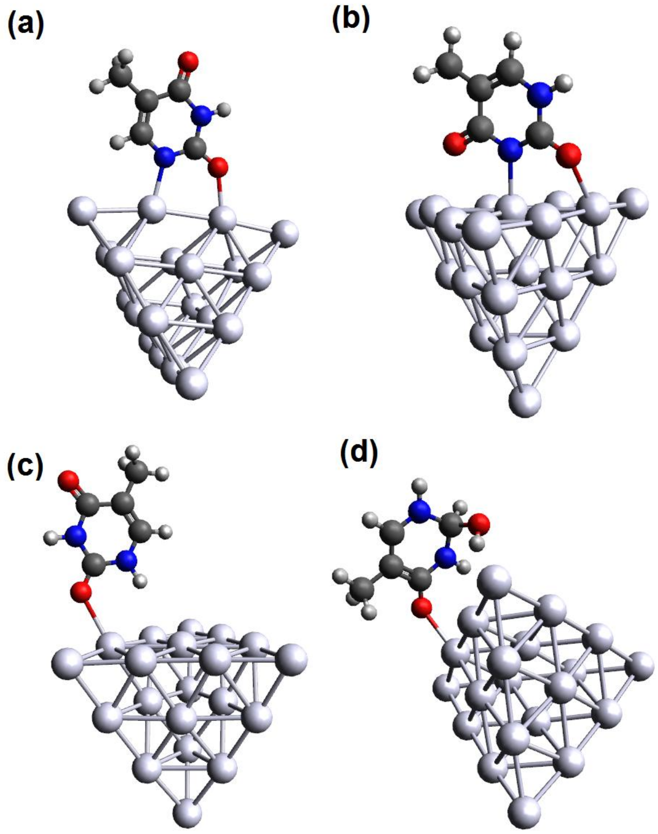

Supplementary Figure 4. Optimized geometries of thymine to the silver tetrahedral structure of (a) N1-E and O2-E, (b) N3-S and O2-E, (c) O2-E, and (d) O4-E

The experimental and calculated frequency modes for each thymine system can be found in supplementary table 4. The top line of each frequency mode represents the location of the

frequency and the bottom line represents the normalized Raman intensity, in which each mode intensity is normalized with respect to the total intensity of the spectrum.

Supplementary Table 4. Experimental and calculated frequency modes (top row of each mode) and intensity ratios (bottom row of each mode) of thymine on multiple binding sites

**Mode**	**Expt**	**N1**	**N3**	**O2**	**O4**
**603-690 cm^{-1}** def C5-CH_3, wag CH_3	603	612	603-657	617	647-690
	0.0576	0.0329	0.0927	0.193	0.0848
**708-756 cm^{-1}** ring-breathing-mode	735	742	754	708-756	744
	0.179	0.156	0.254	0.128	0.0689
**795-841 cm^{-1}** def C5-C6-N1	795	808	799	783-841	788
	0.109	0.167	0.0780	0.111	0.0879
**892-953 cm^{-1}** bend C-O	927	940	905	916-953	892
	0.0319	0.0163	0.00312	0.0408	0.0493
**944-1073 cm^{-1}** def N1-C2-N3, str C5-C6	983	978-1001	988-1073	1017-1070	944-1053
	0.0690	0.0781	0.0284	0.0286	0.0525
**1119-1200 cm^{-1}** str C5-CH_3, C5-C6, C6-N1	1149	1119	1137-1142	1139	1126-1200
	0.0192	0.111	0.0371	0.0234	0.0321
**1193-1262 cm^{-1}** rock CH_3, tor CH_3	1238	1193-1228	1246	1209	1231-1262
	0.0222	0.0588	0.0416	0.0210	0.0725
**1279-1349 cm^{-1}** C5-CH_3	1348	1300-1326	1279-1337	1332-1344	1349
	0.202	0.172	0.0778	0.124	0.0598
**1375-1429 cm^{-1}** bend C5-CH_3, N3-H	1386	1385	1375	1389	1387-1429
	0.320	0.0297	0.0222	0.00841	0.0404
**1391-1452 cm^{-1}** bend C6-N1, rock CH_3	1403	1395	1391	1398	1452
	0.0354	0.0154	0.0442	0.00802	0.0703
**1410-1493 cm^{-1}** bend C5-CH_3	1458	1431-1452	1410	1424	1471-1493
	0.00935	0.0462	0.0653	0.103	0.0193
**1457-1520 cm^{-1}** def C5-C4-N3, wag CH_3	1475	1503	1458	1457	1520
	0.0158	0.0218	0.102	0.0253	0.0299
**1464-1698 cm^{-1}** str C2-O2	1656	1564-1601	1652	1629-1658	1607-1698
	0.218	0.0920	0.154	0.186	0.332

S2. Surface image and characterization of silver films

The SERS substrates used for experimental measurements are random silver films (RSFs) in which an electron beam evaporation system is used to deposit 30 nm of silver onto a silicon substrate. At this thickness, the silver nanoparticles merge together to become irregular shaped islands (Figure S5). The resulting SERS substrates generate consistent electromagnetic field enhancement across a broad excitation wavelength range. The silver films have a face centered cubic (111) surface which is represented by the silver 20 atom tetrahedral structure surface side.

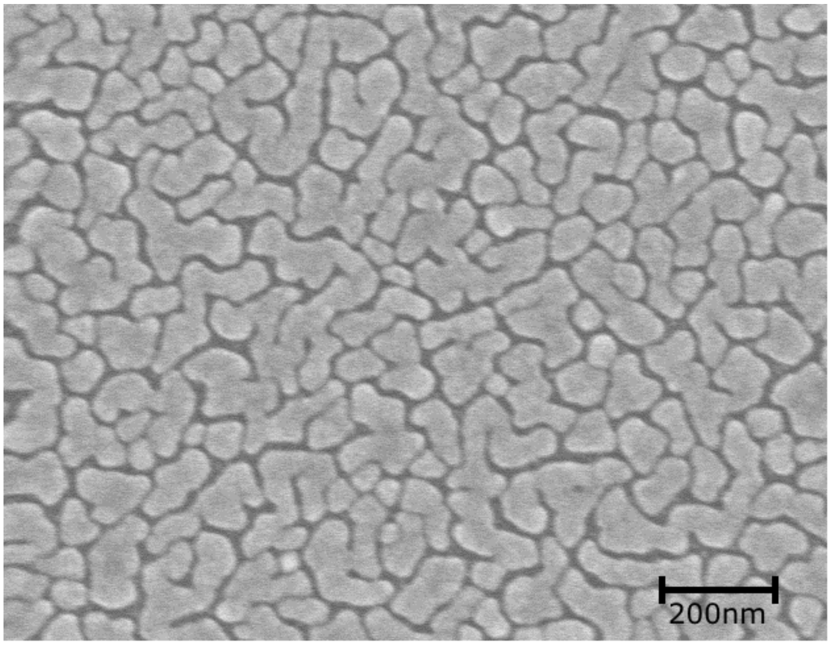

Supplementary Figure 5. Scanning electron microscope image of random silver films

Random silver films have a broad extinction profile in the visible range which has a weak surface plasmon resonance in the near infrared range. Thus, the electromagnetic effect has a minimal effect on the measured Raman spectra. The extinction profile for silver films is shown

in Figure S6, in which two excitation wavelengths (514nm and 785nm) are displayed with their corresponding Raman frequency modes.

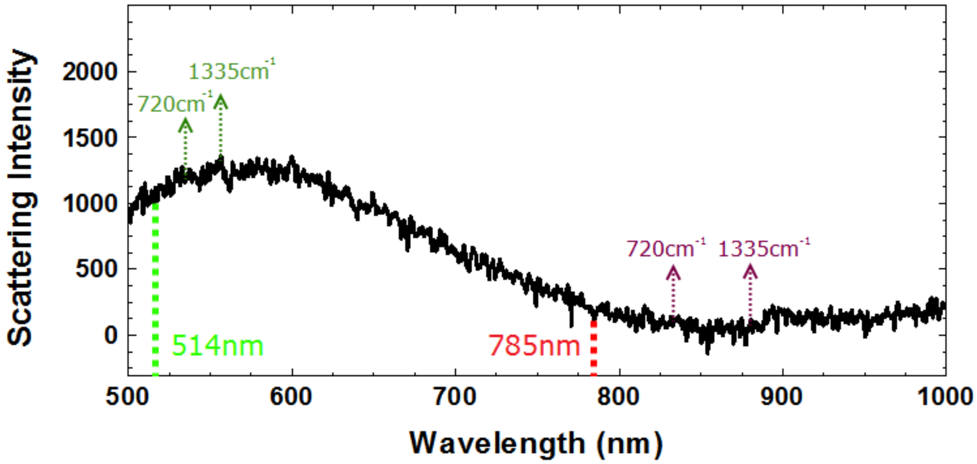

Supplementary Figure 6. Extinction spectrum of silver films showing two excitation wavelengths and the corresponding Raman shifts

While an excitation wavelength of 514nm shows a slight increase in the 1335cm^{-1} mode compared to the 720cm^{-1} mode, the frequency modes excited by the 785nm wavelength show consistent enhancement based on the extinction profile.

To further demonstrate the consistency of the electromagnetic field enhancement across the substrate, Raman maps were acquired of 1 mM of adenine dissolved on random silver films at excitation wavelengths of 514nm and 785nm. An area of approximately 50 microns by 50 microns was analyzed via a raster scan with spot sizes of approximately 10 by 10 microns. For analysis, the peak intensity ratio of the single stretching mode (~1335cm^{-1}) to the ring breathing mode (~720cm^{-1}) was calculated at each spot and a colored Raman map based on the intensity ratio was plotted. Figure S7 shows the resulting Raman maps for 514nm (blue) and 785nm (red).

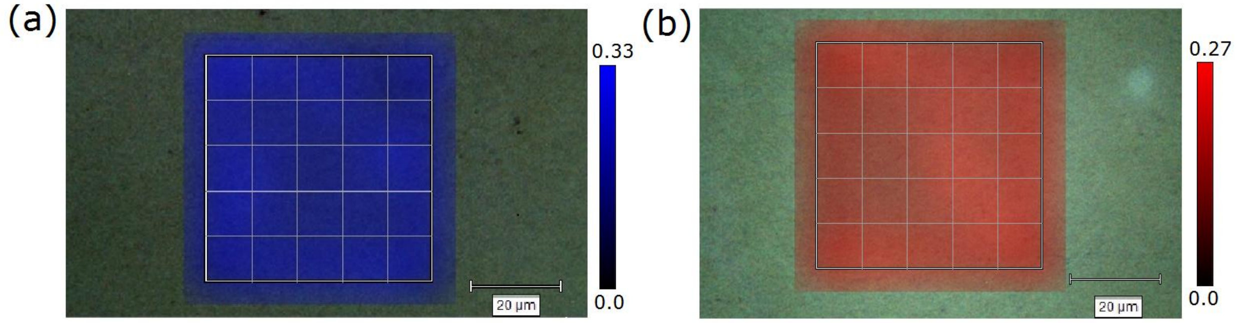

Supplementary Figure 7. Raman map of 50 microns by 50 microns area of adenine functionalized on random silver films for excitation wavelengths of (a) 514nm and (b) 785nm

The Raman maps show consistent peak intensity ratio at each spot along the map for both wavelengths, with an average 1335cm^{-1} to 720cm^{-1} ratio of 0.29 ($\sigma = 0.020$) for 514nm and 0.23 ($\sigma = 0.019$) for 785nm. Thus, the electromagnetic field enhancement effect is fairly consistent across the substrate.

S3. Mode assignments for SRCS process

Before performing the SRCS process, the calculated vibrational frequency modes are aligned with experimental measurements and are normalized with respect to the total Raman intensity of the system. The computed vibrational frequencies are slightly off-set from each other because the frequency modes for each system vary slightly in location dependent on the orientation of the molecule with respect to the surface, as the molecular strain changes the way in which the system vibrates and leads to small shifts in the vibrational frequencies. As an example, the adenine silver systems (A-N1, A-N3, A-N7, A-N9, A-NH_2,N7) have different calculated frequencies for the ring-breathing-mode (RBM) that are determined by the molecular vibrations. GaussView is used to visualize the modes, display the force displacements and appropriately

assign each frequency value with the corresponding mode. The frequencies for A-N1, A-N3, A-N7, A-N9, and A-NH_2,N7 have RBM mode locations of 711cm^{-1}, 698cm^{-1}, 714cm^{-1}, 708cm^{-1} and 716cm^{-1}, respectively. The minor variation between the vibrational frequencies for the RBM is caused by the molecular strain of the system which modulates the bond force constants of the structure. Thus, despite the difference in frequency values, the frequencies from 698-716cm^{-1} are assigned to the RBM by visualizing the force displacements of the modes. The experimental measurements have the RBM slightly red-shifted compared to the simulated measurements due to the scaling factor used in the calculations, with the experimental RBM band ranging from 715cm^{-1} to 743cm^{-1}. The strongest 10 to 12 frequency modes for each nucleic acid, such as the RBM, are selected for analysis using GaussView to visualize the force displacement vectors. The list of modes can be found in supplementary section 1.

S4. Coefficient of Determination Calculations for Single Binding Sites

To compare the coefficient of determinations for single binding sites to the SRCS optimized results, the coefficient of determination for each single atom binding site (e.g. A-N1 compared to experimental measurement) is calculated. As described in the main text, the experimental Raman spectrum mode for a nucleic acid NA and a mode of *i* is defined as E_i^{NA} and the simulated Raman spectrum mode for a nucleic acid NA, a binding site of b, and a mode of *i*, is defined as $S_{i,b}^{NA}(v)$. Thus, the coefficient of determination (r^2) is defined as:

$$r^2 = \left(\frac{\sum_{m=1}^{M} E_m^{NA} S_{m,b}^{NA} - \sum_{m=1}^{M} E_m^{NA} \sum_{m=1}^{M} S_{m,b}^{NA}}{\sqrt{\{\sum_{m=1}^{M} (E_m^{NA})^2 - (\sum_{m=1}^{M} E_m^{NA})^2\} \left\{\sum_{m=1}^{M} (S_{m,b}^{NA})^2 - (\sum_{m=1}^{M} S_{m,b}^{NA})^2\right\}}} \right)^2$$

Here, the coefficient of determination represents the correlation of the simulated Raman spectrum of a single binding site to the experimental Raman spectrum, with a value $r^2 = 1$ constituting perfect correlation. The residuals for each mode are shown in Figure S7, determined by the equation $\varepsilon = \left|E_m^{NA} - S_{m,b}^{NA}\right|$ for each mode m. The bar plots represent the absolute deviation of the simulated mode with respect to the experimental mode and the dotted line shows the mean of the absolute deviation. The scatter plots display the relative deviation for each mode compared to the experimental result (y=0) with the coefficient of determination (r^2) displayed on the plot. The coefficient of determination ranges from very poor (A-NH_2,N7, 0.022) to fairly strong (G-N3, 0.78), with the N3 binding atom showing decent correlation for adenine, cytosine, and guanine. Thymine is very ambiguous, with each atom showing a below average coefficient

of determination.

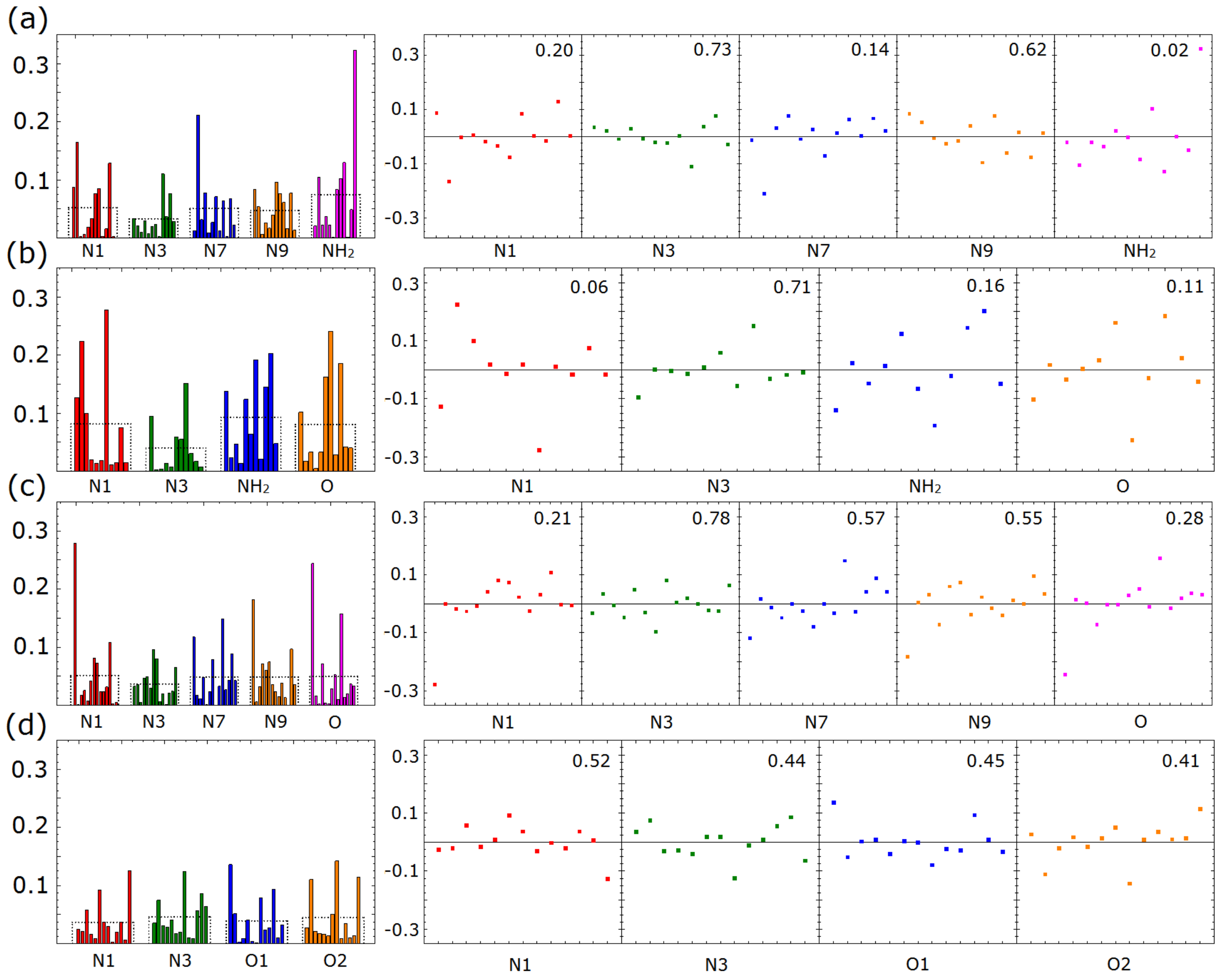

Supplementary Figure 8. The absolute deviation of the simulated normalized Raman mode intensities for (a) adenine, (b) cytosine, (c) guanine, and (d) thymine. Each individual bar represents a mode, starting from the shortest (~600cm^{-1}) to the longest (~1700cm^{-1}) frequency mode. The dotted line represents the average deviation for all modes in that system. Residual plots are shown for each individual system with the coefficient of determination stated in the inset.

Despite the above average correlation for A-N3, C-N3, and G-N3, there are still some simulated modes in these systems that show significant deviation from the experimental values. For example, the C-N stretching mode for A-N3 is greatly enhanced in experimental measurements, but significantly reduced in the simulation results. The C-NH_2 stretching mode for C-N3 is strong in simulations, but very weak in the experimental spectra. Additionally, the C-NH_2 bending mode is absent in the C-N3 simulations. These discrepancies reveal that there is more than one binding site responsible for the Raman signatures in experimental measurements and that the experimental measurements are superimposed spectra of the possible binding sites. To improve the coefficients of determination, the SRCS process is performed in which the coefficient of determination is maximized by finding the optimal weighted coefficient constants for each nucleic acid (main text).

S5. Optimized geometrical Cartesian coordinates for nucleic acid-silver composites

Table S5: Ade-N1 Geometrical Coordinates

Ade-N1			
Ag	1.29690	0.03873	-2.87825
Ag	1.28533	2.45649	-1.48941
Ag	-1.12755	0.88334	-1.53540
Ag	1.28532	2.45649	1.48941
Ag	-1.12755	0.88335	1.53540
Ag	1.25132	4.86827	-0.00000
Ag	-1.16526	3.36809	-0.00001
Ag	1.29689	0.03874	2.87825
Ag	-1.18745	-1.67346	2.91813
Ag	1.19781	-2.45103	4.23175
Ag	2.02324	-0.01512	0.00000
Ag	1.25348	-2.54280	-1.39397
Ag	-1.15587	-1.77359	0.00000
Ag	1.25347	-2.54279	1.39398
Ag	-1.18744	-1.67347	-2.91813
Ag	1.19783	-2.45104	-4.23174
Ag	-3.45934	1.74838	-0.00001
Ag	-3.47520	-0.84523	-1.50477
Ag	-5.71781	0.03889	-0.00001
Ag	-3.47521	-0.84522	1.50476

N	4.51053	-0.24396	0.00001
C	5.38690	0.80829	0.00001
C	4.99849	-1.52392	0.00000
N	6.29046	-1.90748	-0.00000
C	7.14630	-0.85178	-0.00000
C	6.77679	0.50965	0.00000
N	7.90812	1.33764	-0.00000
N	8.53498	-0.84613	-0.00001
C	8.93896	0.49225	-0.00001
H	9.12914	-1.66497	-0.00001
H	9.97822	0.78350	-0.00001
N	4.93533	2.08849	0.00001
H	5.60276	2.84888	0.00001
H	3.94074	2.28704	0.00001
H	4.25206	-2.31082	0.00000

Table S6: Ade-N3 Geometrical Coordinates

**Ade-N3**			
Ag	1.53244	0.57607	-2.66271
Ag	1.22591	2.76096	-0.96214
Ag	-1.04470	1.05854	-1.45515
Ag	0.97822	2.33028	1.97703
Ag	-1.29321	0.61213	1.55295
Ag	0.87181	4.92610	0.83662
Ag	-1.40307	3.28735	0.39746
Ag	1.03660	-0.25465	3.01734
Ag	-1.31124	-2.11418	2.57493
Ag	1.00589	-2.92272	3.99113
Ag	2.08186	0.15336	0.24910
Ag	1.55586	-2.18499	-1.54954
Ag	-1.03030	-1.79254	-0.31460
Ag	1.30870	-2.58383	1.19117
Ag	-0.80405	-1.27744	-3.17498
Ag	1.74195	-1.70731	-4.33968
Ag	-3.55783	1.54235	-0.04678
Ag	-3.26307	-0.79837	-1.89375
Ag	-5.68516	-0.28250	-0.50139
Ag	-3.52153	-1.22859	1.07661
N	6.28951	1.90806	-0.06212
C	7.18636	0.87397	-0.22609
C	5.04639	1.65283	0.72681
N	4.39431	0.36185	0.39857
C	5.30477	-0.59390	0.25389
C	6.72893	-0.40308	0.10331
N	7.38509	-1.64460	-0.08364
N	5.14067	-1.97086	0.13135
C	6.41668	-2.54298	-0.05545

H	4.25120	-2.45752	0.17728
H	6.55312	-3.60934	-0.15761
N	8.40588	1.09993	-0.80146
H	8.97985	0.29326	-1.01259
H	8.81381	2.02246	-0.85120
H	6.34134	2.71718	-0.67210
H	4.35173	2.47348	0.53590
H	5.32320	1.65673	1.79534

Table S7: Ade-N7 Geometrical Coordinates

Ade-N7			
Ag	1.28616	0.17190	-2.87574
Ag	1.11575	2.56498	-1.48363
Ag	-1.18949	0.84134	-1.54128
Ag	1.11574	2.56496	1.48367
Ag	-1.18950	0.84132	1.54128
Ag	0.89442	4.97384	0.00004
Ag	-1.39803	3.30577	0.00002
Ag	1.28614	0.17186	2.87575
Ag	-1.07048	-1.72295	2.91348
Ag	1.35715	-2.32939	4.22892
Ag	2.21596	0.14051	0.00002
Ag	1.43061	-2.37402	-1.39091
Ag	-1.04508	-1.81098	-0.00002
Ag	1.43060	-2.37404	1.39089
Ag	-1.07045	-1.72291	-2.91350
Ag	1.35719	-2.32934	-4.22893
Ag	-3.57309	1.53088	-0.00000
Ag	-3.41478	-1.05490	-1.50608
Ag	-5.71217	-0.33742	-0.00003
Ag	-3.41479	-1.05492	1.50605
N	7.31201	-1.80439	-0.00001
C	5.99866	-1.39981	-0.00000
C	8.30495	-0.93419	-0.00002
N	8.06666	0.44621	-0.00002
C	6.77831	0.89089	-0.00001
C	5.67131	-0.00971	0.00000
N	4.47767	0.65158	0.00001
N	6.29212	2.16920	-0.00000
C	4.78579	2.10720	0.00001
H	6.82461	3.02727	-0.00000
N	5.03306	-2.37114	0.00000
H	4.04630	-2.13685	0.00001
H	5.31586	-3.34186	-0.00000
H	9.33691	-1.25875	-0.00003
H	8.85235	1.08835	-0.00002
H	4.38131	2.61064	0.89351

H	4.38130	2.61065	-0.89347

Table S8: Ade-N9 Geometrical Coordinates

Ade-N9			
Ag	-0.85636	2.12758	2.31635
Ag	-0.34826	3.30215	-0.17964
Ag	1.47471	1.29446	0.82245
Ag	-0.69530	1.70336	-2.69253
Ag	1.14543	-0.31406	-1.73988
Ag	0.22370	4.38826	-2.73680
Ag	2.07518	2.45821	-1.78368
Ag	-1.53630	-0.96086	-2.57081
Ag	0.28046	-3.09139	-1.47114
Ag	-2.33635	-3.67745	-2.33618
Ag	-1.73737	0.71070	-0.10537
Ag	-1.73371	-0.75650	2.50367
Ag	0.50270	-1.58837	0.96671
Ag	-2.21772	-2.18594	0.11414
Ag	1.01891	0.01146	3.39349
Ag	-1.29864	0.85250	4.80583
Ag	3.79076	0.47569	-0.77533
Ag	3.20921	-0.81325	1.86352
Ag	5.44116	-1.57059	0.27649
Ag	2.85911	-2.38704	-0.63241
N	-7.25228	-1.61600	0.07215
C	-7.51476	-0.25613	0.02565
C	-5.94656	-2.13549	0.05192
N	-4.86576	-1.39675	-0.01040
C	-5.07027	-0.02977	-0.05985
C	-6.38320	0.56230	-0.04273
N	-6.27482	1.94117	-0.10274
N	-4.15569	0.97341	-0.12970
C	-4.92918	2.12877	-0.15232
H	-4.47816	3.10863	-0.20467
N	-8.78600	0.22466	0.04557
H	-8.89520	1.23186	0.00868
H	-9.61103	-0.35660	0.09414
H	-5.87277	-3.21646	0.09043
H	-8.01922	-2.27981	0.12221

Table S9: Ade-NH_2,N7 Geometrical Coordinates

Ade-NH_2,N7			
Ag	-0.63006	-3.22619	-0.86830
Ag	0.29471	-2.94075	1.74943
Ag	1.53705	-1.42967	-0.38383
Ag	-0.06064	-0.45647	3.34855
Ag	1.18303	1.06355	1.20367

Ag	1.39555	-2.65882	4.36803
Ag	2.66541	-1.18327	2.31732
Ag	-1.40068	1.78889	2.28691
Ag	-0.11525	3.38708	0.01523
Ag	-2.50169	4.17489	1.26911
Ag	-1.55418	-0.75664	0.90666
Ag	-2.21924	-0.89667	-1.93691
Ag	0.05761	1.03166	-1.63328
Ag	-2.51461	1.86318	-0.51934
Ag	0.39273	-1.40573	-3.09598
Ag	-1.71951	-3.22020	-3.50909
Ag	3.81108	0.29774	0.21154
Ag	2.69641	0.14186	-2.56245
Ag	4.93499	1.77935	-1.94433
Ag	2.42567	2.65665	-0.93818
N	-5.88009	-1.98976	0.30762
C	-4.91671	-1.05909	-0.04875
C	-7.18473	-1.63877	0.38048
N	-7.73497	-0.41824	0.13139
C	-6.78958	0.49026	-0.20513
C	-5.39647	0.26827	-0.30135
N	-4.76385	1.46765	-0.67577
N	-6.97769	1.83404	-0.53443
C	-5.73537	2.37649	-0.80672
N	-3.61979	-1.45614	-0.16251
H	-7.87222	2.30582	-0.56267
H	-5.58845	3.40964	-1.07940
H	-7.87625	-2.42481	0.66771
H	-3.57529	-2.46691	0.01559

Table S10: Cyto-N1 Geometrical Coordinates

Cyto-N1			
Ag	1.39670	0.05839	-2.89680
Ag	1.40179	2.48495	-1.49965
Ag	-0.99772	0.87484	-1.52037
Ag	1.40178	2.48499	1.49957
Ag	-0.99775	0.87491	1.52038
Ag	1.29456	4.89937	-0.00007
Ag	-1.07187	3.35659	-0.00005
Ag	1.39669	0.05847	2.89679
Ag	-1.07106	-1.68641	2.91689
Ag	1.32527	-2.44965	4.22865
Ag	2.02810	0.01215	-0.00000
Ag	1.37833	-2.53708	-1.39589
Ag	-1.03852	-1.78824	0.00003
Ag	1.37834	-2.53704	1.39596
Ag	-1.07106	-1.68650	-2.91684
Ag	1.32527	-2.44978	-4.22859

Ag	-3.34990	1.72156	-0.00003
Ag	-3.35671	-0.86537	-1.50477
Ag	-5.60598	-0.01666	-0.00001
Ag	-3.35672	-0.86533	1.50479
C	5.31738	0.89211	-0.00001
C	5.11818	-1.39477	0.00004
C	7.27849	-0.33872	0.00003
C	6.50314	-1.53682	0.00005
N	4.49610	-0.17703	0.00001
N	8.64593	-0.34054	0.00004
N	6.66501	0.87894	-0.00001
O	4.70981	2.12039	-0.00005
H	9.17381	-1.20057	0.00006
H	4.46026	-2.25863	0.00005
H	6.96539	-2.51700	0.00007
H	9.13332	0.54526	0.00002
H	5.38763	2.83168	-0.00006

Table S11: Cyto-N3 Geometrical Coordinates

**Cyto-N3**			
Ag	1.65228	-0.51937	-2.70103
Ag	0.20608	1.87799	-2.78137
Ag	-1.06179	-0.50614	-1.70064
Ag	-0.35014	3.40697	-0.25344
Ag	-1.62533	1.05525	0.88610
Ag	-1.31327	4.27100	-2.77761
Ag	-2.60053	1.98056	-1.69934
Ag	0.55497	2.43235	2.22217
Ag	-0.71853	-0.03982	3.42191
Ag	1.41068	1.35157	4.69361
Ag	1.41569	1.08131	-0.22215
Ag	2.79579	-1.46821	-0.04816
Ag	0.03917	-1.54158	0.98672
Ag	2.08086	-0.16867	2.38812
Ag	0.46605	-2.98310	-1.43783
Ag	3.06732	-2.98079	-2.49514
Ag	-3.75454	-0.32405	-0.58040
Ag	-2.16249	-2.89429	-0.47152
Ag	-4.81256	-2.67060	0.58669
Ag	-2.72755	-1.39464	2.02752
C	5.07597	0.90017	-0.24399
C	7.50583	0.92828	0.11515
C	6.26574	-1.13204	0.09169
C	7.53087	-0.43945	0.21996
N	6.31917	1.58093	-0.10631
N	6.21644	-2.49004	0.18343
N	5.10659	-0.47840	-0.12083
O	4.03949	1.57797	-0.46049

H	7.04515	-3.04196	0.35235
H	8.39854	1.53838	0.19754
H	8.45727	-0.97306	0.39025
H	5.32407	-2.95830	0.06503
H	6.27138	2.59182	-0.19011

Table S12: Cyto-NH_2 Geometrical Coordinates

**Cyto-NH_2**			
Ag	-1.19971	1.01905	2.82070
Ag	-0.61051	3.07642	1.02308
Ag	1.28908	0.93713	1.35956
Ag	-0.73030	2.55960	-1.92550
Ag	1.16974	0.41075	-1.64908
Ag	0.01554	5.08808	-0.87699
Ag	1.93393	3.01798	-0.58593
Ag	-1.41347	0.01972	-2.87592
Ag	0.51752	-2.28181	-2.56849
Ag	-2.03455	-2.60808	-3.75525
Ag	-1.97102	0.63766	-0.02625
Ag	-1.92135	-1.69878	1.79708
Ag	0.56024	-1.83753	0.31579
Ag	-2.01533	-2.18480	-0.95076
Ag	0.72670	-1.27978	3.17155
Ag	-1.73277	-1.15195	4.57321
Ag	3.70981	0.86470	-0.27405
Ag	3.09787	-1.34226	1.66148
Ag	5.43821	-1.36527	0.05218
Ag	2.98817	-1.85601	-1.29611
C	-6.60541	-1.29290	0.02068
C	-7.84447	0.82777	-0.00273
C	-5.39942	0.90009	0.00114
C	-6.70132	1.56998	-0.00655
N	-7.80506	-0.55955	0.00912
N	-4.22035	1.46809	-0.00915
N	-5.45848	-0.50415	0.02086
O	-6.58874	-2.54085	0.02945
H	-8.83022	1.27933	-0.00967
H	-6.73965	2.65186	-0.01713
H	-8.65225	-1.11576	0.00975
H	-4.56936	-1.00874	0.03621
H	-4.24486	2.49228	-0.01984

Table S13: Cyto-O Geometrical Coordinates

**Cyto-O**			
Ag	-1.34594	0.38885	2.91278
Ag	-0.90479	2.79111	1.51726
Ag	1.14674	0.75426	1.51654

Ag	-0.90479	2.79110	-1.51728
Ag	1.14674	0.75425	-1.51655
Ag	-0.39767	5.13375	-0.00002
Ag	1.66433	3.19280	-0.00001
Ag	-1.34594	0.38883	-2.91278
Ag	0.77609	-1.78320	-2.91605
Ag	-1.71966	-2.10595	-4.22554
Ag	-1.79967	0.44357	-0.00000
Ag	-1.78205	-2.17135	1.39643
Ag	0.74585	-1.88325	0.00001
Ag	-1.78205	-2.17135	-1.39641
Ag	0.77609	-1.78318	2.91606
Ag	-1.71965	-2.10593	4.22556
Ag	3.61120	1.17207	-0.00000
Ag	3.17519	-1.37503	1.50936
Ag	5.53281	-0.93360	0.00000
Ag	3.17519	-1.37504	-1.50935
C	-5.31327	0.37216	0.00000
C	-6.38452	-1.83509	-0.00000
C	-7.66665	0.19242	0.00000
C	-7.62932	-1.25582	-0.00000
N	-5.26025	-1.05173	-0.00000
N	-8.86627	0.84975	0.00000
N	-6.55931	0.95427	0.00000
O	-4.22549	1.01693	0.00000
H	-9.74766	0.35845	0.00000
H	-6.23426	-2.90984	-0.00001
H	-8.53061	-1.85572	-0.00000
H	-8.85160	1.86156	0.00001
H	-4.32489	-1.46519	-0.00000

Table S14: Gua-N1,O Geometrical Coordinates

Gua-N1,O			
Ag	-0.43337	2.93087	-1.49614
Ag	-1.22746	0.63123	-2.89815
Ag	1.29151	0.62094	-1.48822
Ag	-1.96854	-1.93033	-1.54396
Ag	0.56340	-1.96466	-0.00005
Ag	-1.85291	-1.73942	-4.34577
Ag	0.57996	-1.84684	-2.87494
Ag	-1.96854	-1.93041	1.54386
Ag	0.57995	-1.84698	2.87486
Ag	-1.85293	-1.73964	4.34567
Ag	-1.74984	0.77837	0.00001
Ag	-0.43337	2.93079	1.49628
Ag	1.29150	0.62086	1.48825
Ag	-1.22748	0.63109	2.89817
Ag	2.16964	2.99211	0.00008

Ag	0.38432	5.19853	0.00013
Ag	3.05193	-1.72638	-1.52223
Ag	3.80962	0.71478	0.00003
Ag	5.45163	-1.60673	-0.00003
Ag	3.05192	-1.72646	1.52215
N	-4.10780	0.54460	0.00001
C	-4.57408	-0.75688	-0.00002
C	-4.98611	1.60557	0.00003
N	-6.34155	1.51575	0.00004
C	-6.78416	0.23836	0.00001
C	-5.98705	-0.93529	-0.00002
N	-6.80239	-2.08258	-0.00005
N	-8.09485	-0.21163	0.00000
C	-8.04551	-1.61626	-0.00003
H	-8.91926	0.37354	0.00002
O	-3.71900	-1.76285	-0.00005
N	-4.45377	2.87075	0.00006
H	-5.08815	3.65590	0.00008
H	-3.45406	3.01511	0.00006
H	-8.93752	-2.22412	-0.00004

Table S15: Gua-N3 Geometrical Coordinates

Gua-N3			
Ag	-1.01713	-2.63338	-1.38976
Ag	-1.01690	-2.63346	1.38977
Ag	1.36204	-1.73207	-0.00016
Ag	-1.28076	-0.09275	2.87687
Ag	1.07974	0.92533	1.53353
Ag	-0.97630	-2.57254	4.22608
Ag	1.34302	-1.61260	2.91787
Ag	-1.44643	2.31215	1.48575
Ag	0.92229	3.40462	0.00003
Ag	-1.60238	4.72040	0.00027
Ag	-2.06755	-0.19934	0.00016
Ag	-1.28122	-0.09258	-2.87667
Ag	1.07949	0.92541	-1.53364
Ag	-1.44667	2.31224	-1.48539
Ag	1.34255	-1.61243	-2.91818
Ag	-0.97698	-2.57230	-4.22607
Ag	3.56799	-0.60239	1.51172
Ag	3.56775	-0.60230	-1.51232
Ag	5.72813	0.45126	-0.00045
Ag	3.33650	1.96955	-0.00021
N	-6.64135	-1.66037	0.00048
C	-7.55782	-0.53524	0.00058
C	-5.25802	-1.60228	0.00037
N	-4.59252	-0.44010	0.00035
C	-5.40981	0.67044	0.00044

C	-6.82045	0.70475	0.00056
N	-7.27735	2.02834	0.00063
N	-5.01178	1.99087	0.00045
C	-6.17553	2.77188	0.00056
H	-4.05236	2.32533	0.00038
O	-8.78497	-0.76283	0.00067
N	-4.57478	-2.78658	0.00028
H	-6.14830	3.85062	0.00059
H	-7.11305	-2.56133	0.00049
H	-5.04347	-3.68095	0.00030
H	-3.55795	-2.76620	0.00020

Table S16: Gua-N7 Geometrical Coordinates

**Gua-N7**			
Ag	1.42287	-1.12438	-2.56488
Ag	0.69063	1.57447	-2.74497
Ag	-1.22814	-0.33011	-1.72544
Ag	0.41229	3.24359	-0.27090
Ag	-1.50809	1.37190	0.81501
Ag	-0.12126	4.29497	-2.84597
Ag	-2.05397	2.47352	-1.83233
Ag	0.89792	2.12493	2.24904
Ag	-1.03968	0.11547	3.39661
Ag	1.32135	0.90637	4.76768
Ag	1.60857	0.54087	-0.13254
Ag	2.08946	-2.32155	0.15846
Ag	-0.59882	-1.54710	0.99628
Ag	1.68397	-0.78119	2.50896
Ag	-0.45669	-3.13535	-1.39599
Ag	2.11169	-3.85411	-2.28030
Ag	-3.83895	0.58610	-0.76175
Ag	-2.99974	-2.30662	-0.55143
Ag	-5.54554	-1.36851	0.35663
Ag	-3.27570	-0.66629	1.91003
N	6.89354	-1.45528	0.12891
C	5.49709	-1.13256	0.04060
C	7.94304	-0.54522	0.09531
N	7.75144	0.76838	-0.02623
C	6.43628	1.12729	-0.11309
C	5.30297	0.28566	-0.08997
N	4.13459	1.05469	-0.20081
N	5.93803	2.41105	-0.24035
C	4.54598	2.31622	-0.28875
H	6.49336	3.25572	-0.28591
O	4.66464	-2.07529	0.08684
H	7.07409	-2.45187	0.22432
N	9.22020	-1.03166	0.19206
H	9.97494	-0.36038	0.16719

H	9.43195	-2.01361	0.28679
H	3.89951	3.17500	-0.38124

Table S17: Gua-N9,N3 Geometrical Coordinates

Gua-N9,N3			
Ag	1.45356	1.76455	2.22088
Ag	0.09068	-0.42436	3.34504
Ag	-1.18319	1.12497	1.22156
Ag	-0.35573	-2.92831	1.80436
Ag	-1.58417	-1.38946	-0.32926
Ag	-1.39108	-2.57585	4.43375
Ag	-2.66922	-1.09962	2.38296
Ag	0.54776	-3.24421	-0.82653
Ag	-0.53735	-1.45598	-3.07428
Ag	1.53666	-3.32475	-3.50595
Ag	1.41424	-0.79480	0.79949
Ag	2.51229	1.83735	-0.58315
Ag	-0.10231	1.00660	-1.65236
Ag	2.09622	-0.98439	-2.01043
Ag	0.14771	3.38649	-0.05659
Ag	2.56787	4.14532	1.16462
Ag	-3.83232	0.36944	0.27824
Ag	-2.42946	2.68881	-0.93631
Ag	-4.97764	1.84592	-1.87041
Ag	-2.78475	0.15437	-2.51136
N	7.00682	1.06401	-0.21042
C	7.18647	-0.34872	0.10743
C	5.81673	1.69275	-0.51446
N	4.66285	1.02964	-0.55797
C	4.74666	-0.33165	-0.28924
C	5.92415	-1.03786	0.04410
N	5.61232	-2.38715	0.24646
N	3.68951	-1.22010	-0.30633
C	4.29299	-2.45034	0.02685
O	8.34166	-0.74660	0.36962
N	5.85068	3.04272	-0.77570
H	4.98210	3.51760	-0.98086
H	6.70488	3.57831	-0.73558
H	3.71654	-3.36115	0.09800
H	7.87629	1.59065	-0.18999

Table S18: Gua-O Geometrical Coordinates

Gua-O			
Ag	0.44479	0.93885	3.12446
Ag	1.51516	-1.52006	2.27460
Ag	-1.26185	-0.87592	1.59748
Ag	2.36068	-1.89278	-0.63012

Ag	-0.44911	-1.31115	-1.42417
Ag	2.55049	-3.97695	1.34492
Ag	-0.07201	-3.34938	0.56060
Ag	1.76951	0.18193	-2.48793
Ag	-1.01336	0.94085	-3.25401
Ag	1.25098	2.32336	-4.27601
Ag	1.14605	0.60096	0.31535
Ag	0.01516	3.15786	1.15542
Ag	-1.74114	1.47193	-0.48779
Ag	0.65326	2.79392	-1.55034
Ag	-2.36424	1.73054	2.33878
Ag	-0.69825	3.43066	3.87953
Ag	-2.71613	-2.65639	-0.15612
Ag	-3.91771	-0.00526	0.75601
Ag	-5.33907	-1.79042	-0.90499
Ag	-3.17061	-0.45829	-2.12903
N	4.99347	0.47026	-0.19735
C	5.58950	-0.82353	-0.30148
C	5.66448	1.65787	0.05913
N	6.98744	1.71434	0.23221
C	7.60322	0.49850	0.13505
C	7.01112	-0.76222	-0.11544
N	7.98678	-1.76905	-0.13128
N	8.95455	0.24338	0.26968
C	9.13321	-1.13913	0.10051
H	9.66747	0.93616	0.45674
O	4.85002	-1.82593	-0.52936
N	4.91804	2.80237	0.13304
H	10.10286	-1.60902	0.15743
H	3.91076	2.80735	0.03149
H	5.40564	3.66698	0.32065
H	3.97924	0.47849	-0.33439

Table S19: Thy-N1,O2 Geometrical Coordinates

Thy-N1,O2			
Ag	-1.16420	-1.67771	2.92686
Ag	1.08509	-2.63609	1.50747
Ag	-1.27098	-1.76821	-0.00000
Ag	2.99569	-0.92675	0.00001
Ag	0.57640	0.19059	-1.51086
Ag	3.12659	-3.84092	0.00001
Ag	1.08510	-2.63609	-1.50745
Ag	2.34209	2.08878	0.00001
Ag	-0.00968	3.01898	-1.50773
Ag	1.54051	4.83627	0.00000
Ag	0.57639	0.19059	1.51086
Ag	-1.73810	1.29022	2.92171
Ag	-1.87084	1.33208	-0.00001

Ag	-0.00969	3.01898	1.50772
Ag	-3.51619	-0.60137	1.41995
Ag	-3.41487	-0.57407	4.25520
Ag	-1.16419	-1.67771	-2.92686
Ag	-3.51618	-0.60137	-1.41997
Ag	-3.41485	-0.57408	-4.25521
Ag	-1.73809	1.29022	-2.92171
C	7.90155	0.54065	-0.00000
C	6.18076	-1.17577	0.00000
C	5.45174	1.07196	0.00000
N	5.14056	-0.25344	0.00001
C	7.52217	-0.87061	-0.00000
O	4.58484	2.02988	0.00001
N	6.79632	1.42881	0.00000
O	9.07583	0.99667	-0.00001
C	8.61889	-1.90541	-0.00001
H	8.20389	-2.92087	-0.00000
H	9.26579	-1.79109	0.87937
H	9.26578	-1.79109	-0.87938
H	5.86350	-2.21589	0.00000
H	7.00628	2.42379	-0.00000

Table S20: Thy-N3,O2 Geometrical Coordinates

Thy-N3,O2			
Ag	-1.77419	2.70294	-0.82453
Ag	-1.72459	0.57368	-2.71501
Ag	0.72235	1.20798	-1.44374
Ag	-1.56690	-2.38531	-1.62925
Ag	0.99465	-1.79230	-0.35785
Ag	-1.93622	-1.76304	-4.33873
Ag	0.60863	-1.24932	-3.14099
Ag	-1.29297	-2.75531	1.29318
Ag	1.31811	-2.14812	2.45592
Ag	-0.86660	-3.03867	4.05481
Ag	-1.52517	0.04447	0.24109
Ag	-1.14970	2.28989	2.12424
Ag	1.15579	0.65150	1.50020
Ag	-1.02563	-0.34509	3.11288
Ag	1.04608	3.37432	0.40895
Ag	-1.26606	4.87507	0.96904
Ag	3.07722	-0.55559	-1.97713
Ag	3.28525	1.75529	-0.11049
Ag	5.51842	-0.01082	-0.65859
Ag	3.48122	-1.06449	0.99804
C	-4.62716	0.72960	-0.00386
C	-6.51698	-0.80381	0.05681
C	-4.23784	-1.65213	0.08663
N	-5.61429	-1.86356	0.09406

C	-6.08581	0.49195	0.00437
O	-3.46246	-2.69676	0.08480
N	-3.78134	-0.37893	0.06540
O	-4.14580	1.90905	-0.07470
C	-7.01359	1.67777	-0.04652
H	-8.06532	1.36800	-0.02922
H	-6.83395	2.26869	-0.95346
H	-6.82818	2.34877	0.80148
H	-7.56803	-1.07156	0.06792
H	-5.92637	-2.82782	0.11561

Table S21: Thy- O2 Geometrical Coordinates

Thy-O2			
Ag	-0.06696	3.06919	-1.46166
Ag	0.89634	2.91147	1.16931
Ag	-1.58554	1.52632	0.49875
Ag	2.10636	0.35624	2.16365
Ag	-0.24437	-1.16831	1.49213
Ag	1.82705	2.62963	3.83254
Ag	-0.55591	1.21081	3.20672
Ag	2.44020	-1.87888	0.43413
Ag	-0.12728	-3.37208	-0.34734
Ag	2.37290	-4.10540	-1.39211
Ag	1.20720	0.58386	-0.57974
Ag	0.12063	0.69921	-3.28795
Ag	-1.39109	-0.95802	-1.42430
Ag	1.27720	-1.69659	-2.37485
Ag	-2.57427	1.58548	-2.23925
Ag	-1.09883	3.13523	-4.09758
Ag	-2.84557	-0.22920	2.45982
Ag	-3.92368	0.00554	-0.34140
Ag	-5.15056	-1.62812	1.61662
Ag	-2.65296	-2.58231	0.62379
C	8.18636	-0.34570	-0.05279
C	6.35161	1.25559	0.02418
C	5.81062	-1.12044	0.22778
N	5.43853	0.21321	0.17937
C	7.69465	1.04253	-0.09491
O	4.98339	-2.06466	0.36538
N	7.17598	-1.33395	0.11026
O	9.38620	-0.69210	-0.14754
C	8.70775	2.14500	-0.26660
H	8.22633	3.12917	-0.28047
H	9.26863	2.01464	-1.20026
H	9.44449	2.12475	0.54583
H	5.91845	2.24981	0.00348
H	7.48699	-2.30223	0.14344
H	4.44079	0.40077	0.28132

Table S22: Thy- O4 Geometrical Coordinates

Thy-O4			
Ag	1.06665	2.85893	-1.12876
Ag	-0.71901	3.26067	0.99513
Ag	-1.40440	1.31508	-1.06689
Ag	-0.99866	1.19425	3.14981
Ag	-1.71242	-0.85308	1.20408
Ag	-2.56443	3.56950	3.11905
Ag	-3.24747	1.63340	1.16720
Ag	0.54012	-1.15838	3.04909
Ag	-0.02667	-3.23919	0.99277
Ag	2.03355	-3.54406	2.87939
Ag	0.92598	0.82989	0.98107
Ag	2.66721	0.32996	-1.27456
Ag	0.33847	-1.32723	-1.12080
Ag	2.56880	-1.60610	0.81761
Ag	0.45985	0.74869	-3.22722
Ag	2.84943	2.31452	-3.27206
Ag	-3.79616	-0.32581	-0.77403
Ag	-1.85059	-0.82027	-3.01077
Ag	-4.21520	-2.34002	-2.71894
Ag	-2.13187	-2.86999	-0.84330
C	5.83023	-0.79031	0.63331
C	7.53105	-0.07369	-0.93209
C	6.58046	1.64148	0.61167
N	7.55913	1.18558	-0.36225
C	6.70447	-1.07766	-0.50191
O	5.64691	2.62054	0.04734
N	5.90325	0.49058	1.17975
O	5.02528	-1.63972	1.14096
C	6.66822	-2.45513	-1.11789
H	7.39914	-2.54232	-1.93093
H	5.67326	-2.68041	-1.52324
H	6.88668	-3.22700	-0.36938
H	8.22608	-0.22878	-1.75222
H	5.28680	0.67036	1.96710
H	8.15963	1.90108	-0.75021
H	7.06748	2.23568	1.39077
H	4.99255	2.19774	-0.55905
